# Stromal proteome expression profile and muscle-invasive bladder cancer research

**DOI:** 10.1186/1475-2867-12-39

**Published:** 2012-08-25

**Authors:** Haitao Niu, Haiping Jiang, Bo Cheng, Xinhui Li, Qian Dong, Leping Shao, Shiguo Liu, Xinsheng Wang

**Affiliations:** 1 Department of Urology, The Affiliated Hospital of Medical College Qingdao University, Qingdao, China; 2 Department of Oncology, The Affiliated Hospital of Medical College Qingdao University, Qingdao, China; 3 Department of Urology, The Central Hospital of Shengli Oil Field, Dondying, China; 4 Department of Pediatric Surgery, The Affiliated Hospital of Medical College Qingdao University, Qingdao, China; 5 Department of Nephrology, The Affiliated Hospital of Medical College Qingdao University, Qingdao, China; 6 Gout Laboratory, The Affiliated Hospital of Medical College, Qingdao University, Qingdao, China

**Keywords:** Muscle-invasive bladder transitional cell carcinoma, Stroma, Proteome, Subcellular pattern, Biomarker panel

## Abstract

**Background:**

To globally characterize the cancer stroma expression profile of muscle-invasive transitional cell carcinoma and to discuss the cancer biology as well as biomarker discovery from stroma. Laser capture micro dissection was used to harvest purified muscle-invasive bladder cancer stromal cells and normal urothelial stromal cells from 4 paired samples. Two-dimensional liquid chromatography tandem mass spectrometry was used to identify the proteome expression profile. The differential proteins were further analyzed using bioinformatics tools and compared with the published literature.

**Results:**

We identified 868/872 commonly expressed proteins and 978 differential proteins from 4 paired cancer and normal stromal samples using laser capture micro dissection coupled with two-dimensional liquid chromatography tandem mass spectrometry. 487/491 proteins uniquely expressed in cancer/normal stroma. Differential proteins were compared with the entire list of the international protein index (IPI), and there were 42/42 gene ontology (GO) terms exhibited as enriched and 8/5 exhibited as depleted in cellular Component, respectively. Significantly altered pathways between cancer/normal stroma mainly include metabolic pathways, ribosome, focal adhesion, etc. Finally, descriptive statistics show that the stromal proteins with extremes of P*I* and MW have the same probability to be a biomarker.

**Conclusions:**

Based on our results, stromal cells are essential component of the cancer, biomarker discovery and network based multi target therapy should consider neoplastic cells itself and corresponding stroma as whole one.

## Introduction

Despite recent advances in surgical techniques, perioperative chemo radiotherapy and the development of molecularly targeted therapies, muscle-invasive bladder transitional cell carcinoma (BTCC) is still a major epidemiological problem whose incidence continues to rise each year [1]. The prognosis of muscle-invasive BTCC is poor and the 5-year disease specific survival after radical cystectomy remains 50–60% [2]. Though some molecular pathogenesis studies on invasive bladder carcinoma have been undertaken successfully on the gene and transcription levels, the carcinogenic mechanism remains to be elucidated. In this regard, using stroma as a sample may be an alterative way to study muscle-invasive BTCC carcinogenesis. However, there has been no report of this item.

As one of the solid tumors, muscle-invasive BTCC are composed of two independent while interactive components: the neoplastic epithelial cells and the surrounding cancer stroma. These two components interactive and in charge of the cancer biology as a functional whole. Therefore, when looking for the initiator and the molecular mechanisms of muscle-invasive BTCC, it has to be kept in mind that a tumor represents an extensive cellular network of neoplastic and stromal cells [3]. In comparison with the cancer cells, stromal cells are much complicated for stroma contains several types of cells. These cells include fibroblasts, blood vessels and immune cells as well as some other components. Till now, it is difficult to separate the stromal component one by one; however the proteome expression profile represents the final outcome of the complex and dynamic interaction of all kinds of stromal cells. Discovery of proteome expression profile that are integral to neoplastic cells specialized stroma may advance our understanding of the cancer biology and yield novel biomarkers and targets for anticancer therapies.

Based on the above research background, we performed comparative proteomics research on stromal cells from muscle-invasive BTCC. Aimed to enlarge the coverage of proteome profile and so to analyze the data from a systematical standpoint, shotgun strategy was employed. Purified stromal cells were obtained by laser capture micro dissection and two dimensional liquid chromatography in conjunction with tandem mass spectrometry (2D-LC-MS/MS) for the direct analysis of complex mixtures was used for the construction of expression profile.

After the construction of expression profile, gene ontology (GO) analysis was performed and the global features of the expression profile as well as biomarker panel discovery were discussed. Aimed to achieve a systematical description of pathway changes and facilitate the network-based multi-target therapy as well as biomarker discovery, a careful analysis of the pathways in the Kyoto Encyclopedia of Genes and Genomes (KEGG) database was conducted with the Array Track software.

## Methods

### Patients and tissue samples

A total of 4 paired muscle-invasive BTCC and normal urothelial samples (confirmed by two individual pathological diagnoses) were obtained from patients treated at the Affiliated Hospital of Medical College Qingdao University immediately after radical cystectomy due to primary invasive bladder cancer. The cancer and the adjacent microscopically normal urothelium (away from 5 cm) samples were rinsed in sterile PBS and snap frozen in liquid nitrogen within 30 min of removal. The research protocol was approved by the Institutional Review Board and informed consent was obtained from patients.

### Laser capture micro dissection

Eight-micrometer sections of freshly prepared tissues were stained with hematoxylin and eosin (H&E) using standard manufacturer’s protocols with minor modifications. Once air-dried, the sections were micro dissected with a Leica AS LMD Laser Capture Micro dissection System (Sunnyvale, CA). To avoid the degradation of protein, we captured the cells within 120 min each cap. Laser capture micro dissected cells were dissolved in lysis buffer (95 mM urea, 4% CHAPS, 40 mM Tris, 65 mM DTT). Samples were solubilized via sonication using 20 second bursts, followed by ice cooling (20 seconds), in a process that was repeated 5 times. After that, the crude tissue extracts were centrifuged for 45 min at 15,000 rpm to remove the undissolved pellets. All samples were stored at −80°C until use.

### Digestion of protein mixture and 2D-LC-MS/MS

Samples prepared by LCM technology were deposited in precipitation solution (50% acetone/50% ethanol/0.1% acetic acid, sample volume: precipitation solution volume = 1:5) for at least 12 h at −20°C. The pellets were washed by 100% acetone and 70% ethanol, then redissolved in 6 mM guanidine-HCl/100 mM Tris (pH 8.3), and the concentrations were measured by Bio-Rad protein assay kit. Next, 200 μg of soluble proteins were reduced with DTT (final concentration 20 mM) and subsequently alkylated with IAA (final concentration 40 mM). After desalting and removal of toludine blue by ultrafiltration with Microcon-10, the protein mixture was incubated overnight at 37°C with trypsin (trypsin: protein mixture = 1:30 w/w; Promega). Two-dimensional high-performance LC separations were performed on a ProteomeX work station (Thermo Finnigan) equipped with two capillary LC pumps. The flow rate of both salt and 4 analytical pumps was at 120 μl/min and was about 1.5 μl/min after split. Nine different salt concentration ranges from 0, 25, 50, 75, 100, 150, 200, 400, and 800 mM ammonium chloride were used for step gradient. The mobile phases used for reverse phase were: A, 0.1% formic acid in water, pH 3.0; B, 0.1% formic acid in acetonitrile. The Finnigan LTQ linear ion trap mass spectrometer was used for the MS/MS experiment with an ion transfer capillary of 160°C and Spray voltage of 3.0 kV. Normalized collision energy was 35.0%. After acquisition of full-scan mass spectra, 10 MS/MS scans were acquired for the next 10 most intense ions using dynamic exclusion. Peptides and proteins were identified using Sequest software (Thermo Finnigan), which uses the MS and MS/MS spectra of peptide ions to search against the publicly available IPI (International Protein Index) database. The spectra for singly charged peptides with a cross-correlation score to a tryptic peptide (*X*corrs) greater than 1.9, the spectra for doubly charged tryptic peptides with *X*corrs of at least 2.2, and the spectra for triply charged tryptic peptides with *X*corrs above 3.75 were accepted as correctly identified according to Sequest software. For all the spectra analyzed, Δ*Cn* values were above o.1.

### GO enrichment/depletion analysis

To take an overview of our comparative proteomics analysis, the cancer/normal stroma specific proteins were categorized as to GO assignments (http://www.geneontology.org), and GOfact software was used to find statistically over- or under-represented GO categories in biological data as the tool for enrichment analysis of our proteome dataset [4]. For enrichment analysis, a test dataset (which is our identified proteins) and a reference set of GO annotation for the complete human proteome were in need. As per instructions on the GOfact webpage, the custom GO annotation for the reference set (of whole IPI human dataset) was created by extracting the GO annotations available for Human IPI IDs from EBI GOA Human 80.0 release. The analysis was done using “hyper geometric test”; the GO terms with P < 0.05 or P < 0.01 were selected as enriched/depleted or significantly enriched/depleted.

### Pathway analysis

To take an overview of our comparative proteomics analysis, the differential proteins were categorized first. The proteins uniquely expressed in cancer or normal stroma were considered as differential expressed. Next, the IPI names of the differentially expressed proteins were converted to SWISS-PROT names for SWISS-PROT was a protein sequence database of low redundancy with high levels of annotation. Array Track software was used for pathway analysis. Array Track offers a simple query interface to retrieve information about human protein expression profile, and provides direct connections to related metabolic and regulatory pathways available from Kyoto Encyclopedia of Genes and Genomes (KEGG) [5]. Most of all, Array Track software can analyze expression profile without the consideration of differential abundant. For statistical analysis, a P value for pathway enrichment was obtained using the hyper geometric test and P <0.05 was considered statistically significant.

### Evidence based biomarker exploration

The proteins that located in KEGG pathways by Array Track were defined as potential biomarkers and the basic physical chemistry of these proteins were further discussed. Descriptive Statistics was used for calculate the distribution of the data. Interquartile-range (IQR) was used to give the basic characteristic of the variability skewness distribution data. Categorical variables were expressed as a frequency and were compared by chi-square. All analysis was performed with SPSS®, version 16.0 and *P* < 0.05 was considered statistically significant.

## Results

### Purify of the cells

**Figure 1:**
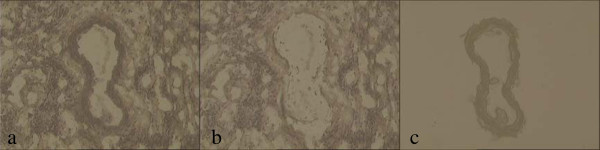
**Harvest the cancer stromal vasculature by LCM.** (**a**) Before LCM; (**b**) after LCM; (**c**) the microdissected stromal vasculature on cap.

Approximately 500, 000 shoots of cancer stromal cells and normal stromal cells from each sample were micro dissected and stored on micro dissection caps at −80°C until lysed. Each cell population was determined to be 98% homogeneous by microscopic visualization of the captured stroma. Figure 1 shows the precision of LCM by a representative harvest of blood vessels which is one of the cancer stromal components.

### Identification of the proteins

We respectively identified 998, 991, 957, 1086 proteins from the 4 cancer stromal samples. Among them, 868 proteins commonly expressed in 4 cancer stromal samples and defined as the overall expression profile of cancer stroma. In parallel, 982; 964; 1022; 979 proteins respectively identified from the 4 normal stromal samples. After data match, 872 proteins commonly expressed in 4 normal stromal samples and defined as overall expression profile of normal stroma. The proteins uniquely detected in cancer versus normal stroma were defined as differentially expressed, vice versa. After comparison, 381 proteins commonly identified in cancer and normal stroma, 978 proteins showed differentially expressed between cancer and normal stroma. To obtain global description of the biological characteristic of invasive bladder cancer stroma, we only analyzed the 978 differentially expressed proteins through comparison of cancer versus normal stromal samples (487 proteins uniquely expressed in cancer stroma and 491 proteins uniquely expressed in normal stroma). The smallest and largest molecular weight (MW) values observed in differential proteins were 6.78 and 986.90 kDa, and the proteins were distributed across a wide isoelectric point (P*I*) range (3.67-11.98).

### GO and GO enrichment/depletion analysis

**Figure 2:**
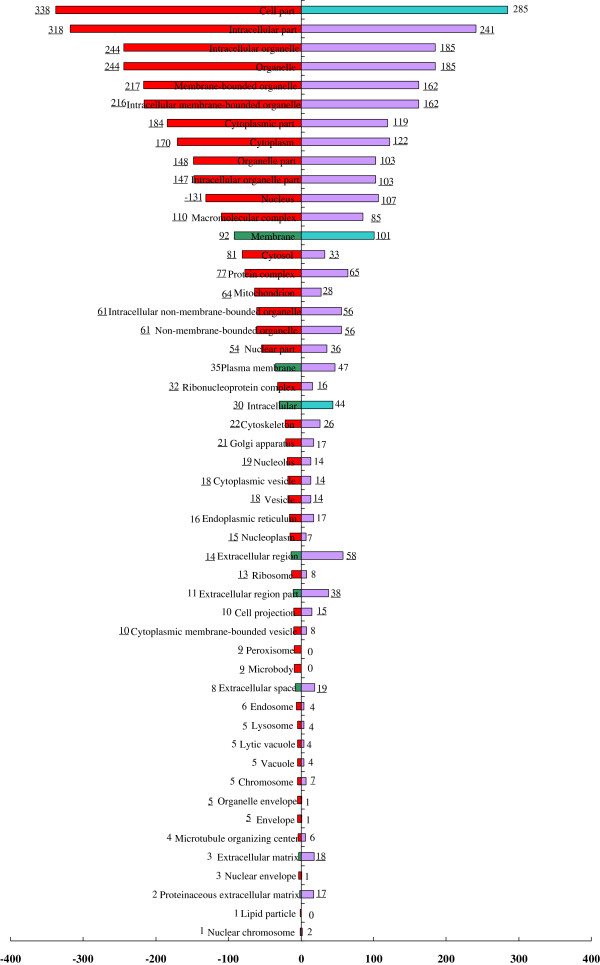
**Enriched/depleted GO celluar component terms for the set of cancer/normal cells specific proteins.** Purple/red indicates enriched terms in normal/cancer stromal cells; light blue/dark green indicates depleted terms in normal/cancer stromal cells. Underline indicates significantly enriched/depleted terms.

After analysis, 347 and 319 of the proteins that specific expressed in cancer and normal stroma own GO cellular component annotation. Compared with the entire list of the IPI (IPI_Human, versions 3.53, 219486 entries for 30786 proteins), there were 42 GO cellular component terms exhibited as enriched and 8 terms exhibited as depleted in cancer stroma. In parallel with this, however, there were 42 terms exhibited as enriched and 5 exhibited as depleted in normal stroma. Figure 2 showed enrichment/depletion analysis of cellular component. The enriched GO cellular component terms mainly include protein complex, cytosol, macromolecular complex, mitchondrion, nuclear part, ribonucleoprotein complex, cytoskeleton, golgi apparatus, ribosome, etc.

### Significant pathways and identification of potential biomarkers

**Figure 3:**
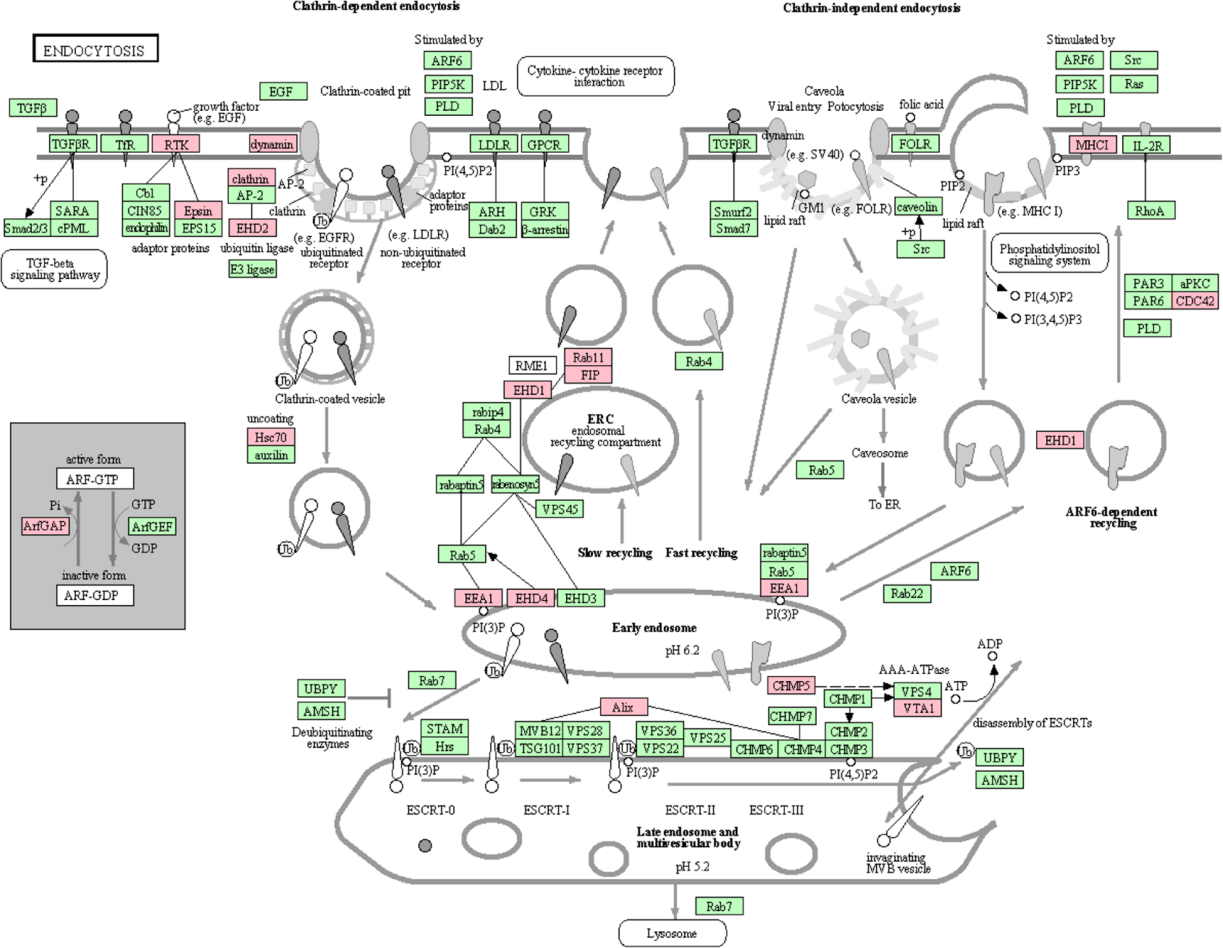
**Representative KEGG pathway change of Endocytosis.** Red indicate the differential proteins located in KEGG pathway.

After conversion, 754 of the 978 differentially expressed proteins with SwissProAcc numbers and were analyzed by Array Track software. After analysis, 409 proteins located at the well-known biological KEGG pathways and defined as potential biomarkers. The significant altered pathways mainly include metabolic pathways, spliceosome, regulation of actin cytoskeleton, ribosome, focal adhesion, Endocytosis, oxidative phosphorylation, etc. Figure 3 showed a typical changed KEGG pathway. Table 1 lists the major altered pathways that include at least 10 differential proteins as well as the proteins specific expressed in cancer stroma and located at metabolic pathways.

**Table 1:** Major altered pathways that include at least 10 differentially expressed proteins, underline indicates the protein specific expressed in cancer stroma

**Pathway**	**Gene name**
Metabolic pathways	ACAA1, ACADSB, ACO2, ACOX1, ACSL5, ACSM1, ACSM3, ADH1B, ADSS, AK2, AKR1B10, ALDH1B1, ALDH4A1, APIP, APRT, ASAH1, ATIC, ATP5I, ATP6V1E1, BCKDHA, BCKDHB, CAT, CBS, CHPF, COMT, COX17, COX5B, CS, CYC1, CYP19A1, DAD1, DBT, DDOST, DGKI, DLAT, DLD, DLST, DUT, FASN, FH, G6PD, GANAB, GNPDA1, GOT2, GRHPR, HADH, HADHB, HIBADH, HK1, HMGCL, HMGCS2, HSD17B4, IDH1, IMPDH2, ITPA, ITPKA, LAP3, MAT2A, NANS, NDUFA10, NDUFA4, NDUFA8, NDUFS4, NDUFS6, NFKB1, NME2, NOS3, OGDH, Cav-1, PAICS, PCK2, PDXK, PFKP, PGD, PGM2, PHPT1, PNPO, POLR2D, PRDX6, SDHB, SPTLC1, SUCLG2, TYMP, UGDH, UQCRB, UQCRC1
Spliceosome	ACIN1, BCAS2, CRNKL1, HNRNPA1, HSPA2, HSPA6, NHP2L1, PUF60, RBM8A, RBMX, SF3A3, SF3B1, SF3B2, SFRS13A, SFRS2, SFRS3, SFRS4, SFRS5, SFRS9, SNRNP200, SNRPB, SNRPC, SNRPD3, SNRPF, SNRPG, TRA2B, U2AF2
Regulation of actin cytoskeleton	ARPC1B, ARPC2, ARPC3, ARPC5, CDC42, CFL2, CRK, ENAH, IQGAP3, ITGA5, ITGA6, ITGAV, ITGB1, ITGB4, MYH14, MYLK, PIK3CB, ROCK2, RRAS2, SSH1, SSH3, WASF2
Ribosome	RPL18, RPL19, RPL22, RPL23, RPL23A, RPL31, RPL38, RPL5, RPL7A, RPL9, RPLP0, RPLP1, RPS10, RPS17, RPS20, RPS25, RPS4X, RPS7
Focal adhesion	CDC42, COL3A1, COL4A2, CRK, CTNNB1, FLNC, FLT1, ITGA5, ITGA6, ITGAV, ITGB1, ITGB4, MYLK, PIK3CB, RAP1B, ROCK2, TNC, TNXB, VTN, VWF
Proteasome	PSMA3, PSMA4, PSMA5, PSMA7, PSMB2, PSMB7, PSMC1, PSMC2, PSMC4, PSMC6, PSMD2, PSMD7, PSME2
Endocytosis	ACAP2, ARFGAP2, CDC42, CHMP5, CLTA, CLTB, DNM2, EEA1, EHD1, EHD2, EHD4, EPN1, FLT1, HLA-C, HSPA2, HSPA6, PDCD6IP, RAB11B, RAB11FIP1, VTA1
Huntington's disease	CASP8, CLTA, CLTB, COX5B, COX7A2, CYC1, CYCS, DCTN1, NDUFA10, NDUFA4, NDUFA8, NDUFS4, NDUFS6, POLR2D, SDHB, SOD1, TAF4, UQCRB, UQCRC1
Alzheimer's disease	ADAM10, APOE, ATP2A3, CAPN1, CASP8, COX5B, COX7A2, CYC1, CYCS, LRP1, NDUFA10, NDUFA4, NDUFA8, NDUFS4, NDUFS6, PPP3R1, SDHB, UQCRB, UQCRC1
Oxidative phosphorylation	ATP5I, ATP6V1E1, COX17, COX5B, COX7A2, CYC1, NDUFA10, NDUFA4, NDUFA8, NDUFS4, NDUFS6, PPA1, SDHB, UQCRB, UQCRC1
Systemic lupus erythematosus	C8G, C9, CTSG, ELANE, H2AFV, H2AFY, HIST1H2AA, HIST1H2BA, HIST2H3A, HIST2H3C, HIST2H3D, HLA-DRB1, SNRPB, SNRPD3, SSB
Valine, leucine and isoleucine degradation	ACAA1, ACADSB, ALDH1B1, BCKDHA, BCKDHB, DBT, DLD, HADH, HADHB, HIBADH, HMGCL, HMGCS2
Pathogenic Escherichia coli infection	ARPC1B, ARPC2, ARPC3, ARPC5, CDC42, CDH1, CTNNB1, CTTN, HCLS1, ITGB1, ROCK2, TUBB3
ECM-receptor interaction	AGRN, COL3A1, COL4A2, ITGA5, ITGA6, ITGAV, ITGB1, ITGB4, TNC, TNXB, VTN, VWF
Complement and coagulation cascades	C4BPA, C8G, C9, CFH, CFI, KNG1, PLG, SERPINC1, SERPING1, VWF
Citrate cycle	ACO2, CS, DLAT, DLD, DLST, FH, IDH1, OGDH, PCK2, SDHB, SUCLG2

### Evidence based biomarker exploration

**Figure 4:**
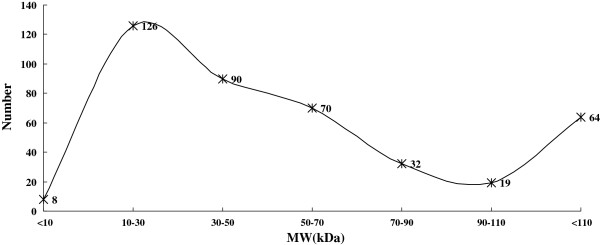
**Distribution of P*I *about the potential biomarkers.** Turning curve shows the global skewness distribution of the data. The data that P*I* ≤ 8 (Skewness = 0.330 SE = 0.146; Kurtosis = 0.425, SE = 0.292, *P*<0.05) and 8<P*I* ≤ 10 (Skewness = 0.211, SE = 0.240; Kurtosis = 0.198, SE = 0.476, *P*<0.05) also showed skewed distribution though looks like normal distribution.

**Figure 5:**
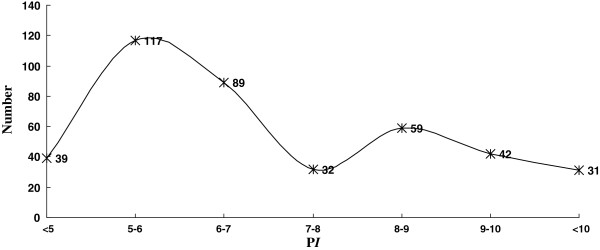
Turning curve shows the global skewness distribution MW about the potential biomarkers.

Array Track defined a total of 409 proteins as potential biomarkers. Figure 4 and Figure 5 show the distribution of P*I* and MW about the potential biomarkers as well as the skewness distribution of the data. When subdivide the data by P*I* equal 9 and MW equal 100 kDa, the IQR was 1.70 for P*I* ≤ 9 and 1.18 for P*I*>9, 33.28 for MW ≤ 100 and 112.07 for MW>100, respectively. Though the data that P*I* ≤ 8 and 8<P*I* ≤10 looked like normal distribution, the pseudomorphism was see through by descriptive statistics. Chi-square test showed that the proteins with extremes of P*I* and MW have the same probability to be a biomarker (Table 2).

**Table 2:** **Chi-square test showed that the proteins had same chance to be a biomarker and irrespective with the P*I*, MW**

**Variable**		**Protein number (n = 754)**		
		**Biomarker (n = 409)**	**Non (n = 345)**	**P value**
P*I*	≤9	336	297	>0.05
	>9	73	48	
MW (kDa)	≤100	326	279	>0.05
	>100 or<10	83	66	

## Discussion

Tissue specific proteomics data can only be generated if the sample investigated consists of homogeneous cell population. Then as the initial step of our study about cancer stroma, LCM is employed to harvest purified stromal cells. After comparison, 978 proteins showed differentially expressed between cancer and normal sample. Up till now, our outcome present the largest stromal proteome expression profile of muscle-invasive BTCC by compared with the published literatures. A significant number of differential proteins have been previously reported functional in carcinogenesis. Elevated expression of ITGB1 and activation of ITGB1-coupled signaling have been implicated in the induction and propagation of a wide variety of human cancers and targeted disruption of ITGB1 has shown to inhibit both the initiation and maintenance of mammary cancer growth in vivo [6]. ROCK2 was overexpressed in hepatocellular carcinoma, and down-regulation of ROCK2 can suppress cancer metastasis and progression [7]. TNC was an extracellular matrix glycoprotein which was frequently up-regulated in a variety of cancers and has been implicated in the modulation of cell migration, proliferation, invasion and angiogenesis [8]. Other examples are TNXB, ARPC2, etc. [9,10]. Though present study offer a list of candidate biomarkers, the results were derived from a small number of cases and should thus be considered preliminary. Aimed to avoid this disadvantage and enhance the scientificalness of the article, we further analyzed the expression profile by GO and pathway analysis.

Proteomics characterization of individual subcellular fundamental patterns (e.g., organelles) can provide valuable information regarding their function in cancer [11]. In addition, this type of approach circumvents analytical issues associated with the complexity of the entire proteome and represents a tractable method for determining the proteome of a given subcellular structure. At a conceptual level, the most complete model of subcellular patterns is probably the GO cellular component ontology. We can imagine easily associating GO terms with most fundamental patterns by checking which organelle markers are assigned to each. Compared with all human proteins deposited on IPI, the GO enrichment/depletion of the subcellular pattern mean the proteins in these categories are over/under presented in this profile and reflect the biologically specific categories of these data. By this way, shotgun strategy can understand carcinogenesis by subcellular level even without consideration of differentially abundant. As shown in Figure 2, our observation indicated that the global over/under represented terms in subcellular pattern of invasive bladder cancer stroma and normal urothelial stroma was quite consistent. Besides the global consistence, the proteins belong to each term were completely different. The different proteins clustered in each same term reflect different molecular networks can maintain the same basic subcellular pattern; meanwhile the dysfunctions of specific subcellular pattern may lead to carcinogenesis. From Figure 2, the main dysfunctional cellular components in our expression profile include mitochondrion, golgi apparatus, ribosome, etc. Many lines of evidence suggest that mitochondrion has a central role in carcinogenesis [12]. An altered ribosome could alter the mRNA pool that is translated, preferentially increasing the translation of oncoproteins or decreasing the translation of cancer suppressing proteins [13]. Golgi apparatus is the central hub for protein sorting and lipid metabolism in the secretory pathway. Despite major advances in elucidating its molecular biology in carcinogenesis, the fundamental question of how the morphogenesis of this organelle is organized on a system level has remained elusive [14]. Though our outcome can provide candidate subcellular pattern for further analysis in muscle-invasvie BTCC stromal study, there are a number of significant challenges remain. Primarily related to the complexity of proteomes, such as the overlapping physical properties of organelles, some proteins may be present in multiple subcellular locations, etc. Major breakthrough must rely on methodological improvements and extensively cooperation.

Cancer is now appreciated as a disease involving dysfunction of multiple biological pathways [15]. Understanding of changes through a proteomics pathway approach can facilitate not only biomarker discovery but also network based multi target therapy. The differential proteins in our practice mainly located at focal adhesion, metabolic pathways, etc. Focal adhesions have emerged as an important therapeutic target in cancer and focal adhesion protein specific siRNAs have been assessed in several different human cancer cell lines. Although siRNAs seem to be relatively efficient in suppressing focal adhesion protein expression, it does not induce a common set of phenotypes in nude mice. This fact indicates that the cancer stroma may play a compensatory role in governing phenotypic responses on loss of focal adhesion protein expression [16]. Alterations in focal adhesion are among the most consistent hallmarks of cancer, while the change of metabolic pathway in stroma offered great thinking space about cancer research. For a long time, metabolic research, limited to cancer cells themselves and called “Warburg effect”. Recently, this kind of traditional view is questioned and has the tendency of substituted by “the reverse Warburg effect.” In this new theory, aggressive cancer cells are “parasites” that use oxidative stress as a “weapon” to extract nutrients from surrounding stromal cells. Oxidative stress in stromal cells induces the autophagic destruction of mitochondria by mitophagy. Then, stromal cells are forced to undergo aerobic glycolysis, and produce energy-rich nutrients to “feed” cancer cells, thus to construct the so-called stromal–epithelial metabolic coupling [17]. As shown in Table 1, 86 differential proteins located at metabolic related pathways, this fact stress the importance of cancer researches aimed on stromal metabolism. These pathway changes stresses that stromal cells are essential component of the cancer, and network based multi target therapy should consider neoplastic cells themselves and corresponding stroma.

Another contribution of pathway analysis is the presentation of candidate biomarkers. Take the metabolic pathways mentioned previously that not only maintain the primary function of stromal cells but also consist of the “hot-spots” in cancer research as examples. 86 proteins located at the well-known KEGG metabolic pathways, 60 proteins uniquely expressed in cancer stromal sample and the remains uniquely expressed in normal stromal sample. Although quantitative information cannot be obtained, specific expressed proteins mean either the proteins only expressed in one tissue or the expression of protein exist significant difference based on same sample amounts. Nowadays, the notion of biomarker panel that composed by the combination of independent, complementary markers has been generally recognized [18]. Based on the former description, we can primarily conclude the proteins as listed in Table 1 might be effective candidates for the biomarker panel research. Besides the few numbers of proteins in these pathways that have been reported in previous literatures, most of them are first present in muscle-invasive BTCC research. Enzyme fatty acid synthase (FASN) that shows cancer specifically expressed in our practice is a key metabolic enzyme in energy homeostasis and has been found up-regulated in different malignant tumors. Several small molecule inhibitors of FASN have now been described or in development for therapeutic use [19]. The roles of these proteins in the BTCC biology deserve further investigation.

In our practice, the largest MW values observed in differential proteins was 986.90 kDa, and the proteins were distributed across a wide P*I* range of 3.67 to 11.95. 409 proteins were defined as potential biomarkers by pathway analysis include 73 proteins that P*I*>9 and 83 proteins that MW>100 kDa or<10 kDa. Further analysis shown these proteins have the same probability to be a biomarker as common proteins. Based on these data, cancer stroma can be the ideal source of biomarkers and the proteins with extremes of P*I* and MW should not be neglected in cancer research. Though the present study performs comparative proteomics analysis of purified muscle-invasive BTCC and corresponding stroma, the number of differential proteins was too much to be validated. Then we are conceiving of gel-based techniques to offer complementary information and to decrease the scope of candidate biomarkers. Aimed to guide selectively gel excision in 2-DE technology, we performed evidence based biomarker exploration. For skewness distribution data the IQR is often the preferred descriptive statistics. After divided the data by the detection range of routine 2-DE, the IQR is 1.70 for PI ≤ 9 and 1.18 for PI>9, 33.28 for MW ≤ 100 and 112.07 for MW>100, respectively. Though the data that PI ≤ 8, 8<PI ≤ 10 looked like normal distribution, the pseudomorphism was see through by Descriptive Statistics. Though the data that PI ≤ 8 and 8<PI ≤ 10 have skewness distribution, they show tendency that biomarkers centered a range of 5 to 8 and 8–10 in the first dimension of the gel. As to the distribution of MW, descriptive statistics can not find some regularity.

## Competing interests

We state that there are no conflicts of interest in this manuscript.

## Authors’ contributions

NHT performed 2D-LC-MS/MS, and wrote the manuscript. JHP performed part of the 2D-LC-MS/MS and made the draft of the manuscript as co-first author. CB performed LCM. LXH carried out the GO analysis. DQ and SLP carried out the pathway analysis. LSG carried out the descriptive statistics on candidate biomarkers. WXS, as the corresponding author, designed the protocol and revised the manuscript. All authors read and approved the final manuscript.
